# Optimization of real-time PCR protocols from lymph node bovine tissue for direct detection of *Mycobacterium tuberculosis* complex

**DOI:** 10.1128/spectrum.00348-23

**Published:** 2023-09-14

**Authors:** Eduardo Vera-Salmoral, Jaime Gómez-Laguna, Ángela Galán-Relaño, Inés Ruedas-Torres, Librado Carrasco, Inmaculada Luque, Belén Huerta, José María Sánchez-Carvajal

**Affiliations:** 1 Department of Anatomy and Comparative Pathology and Toxicology, Pathology and Immunology Group (UCO-PIG), UIC Zoonosis y Enfermedades Emergentes ENZOEM, University of Córdoba, International Excellence Agrifood Campus ‘CeiA3’, Córdoba, Spain; 2 Department of Animal Health, UIC Zoonosis y Enfermedades Emergentes ENZOEM, International Excellence Agrifood Campus ‘CeiA3’, University of Córdoba, Córdoba, Spain; 3 Institute of Virology and Immunology IVI, Sensemattstrasse, Mittelhäusern, Switzerland; University of California, Davis, San Bernardino, California, USA

**Keywords:** direct real-time PCR, extraction protocol, fresh tissue, IS*6110*, IS*4*, mpb70, bovine tuberculosis

## Abstract

Bovine tuberculosis (bTB) is a zoonotic disease and a global health problem that is subjected to obligatory eradication programs in the European Union. Microbiological culture is an imperfect technique for bTB diagnosis. This study aims to compare and validate two DNA isolation protocols and three different specific DNA targets, IS6110, IS4, and mpb70, to confirm *Mycobacterium tuberculosis* complex (MTC) infection by real-time PCR directly from fresh tissue samples. Fresh lymph node samples were collected from 81 cattle carcasses at the slaughterhouse. A comparison of both extraction protocols was performed with IS*6110*-real-time PCR, showing an adjusted sensitivity (SE) of 78.34% and 95.9% for protocols 1 and 2, respectively, while the specificity (SP) was 100% in both cases. Afterward, the comparison between IS4 and mpb70 targets was performed from the samples extracted with protocol 2, obtaining an adjusted SE of 90.87% and 83.3%, respectively, and an SP of 100% in both cases. The positive likelihood ratio was ∞ for the three targets, and the negative likelihood ratio was 0.04, 0.091, and 0.16 for IS6110, IS4, and mpb70, respectively. Negative predictive values were ≥90%, ≥85%, and ≥80% for real-time PCR targeting IS*6110*, IS4, and mpb70, respectively, when the true prevalence is ≤60%, and the positive predictive value is 100% in any scenario of true prevalence. According to these results, the DNA extraction protocol 2 and real-time PCR targeting IS*6110* or IS4 could be potential first-choice molecular assays to detect MTC directly in fresh bovine tissue samples.

**Importance:**

Bovine tuberculosis (bTB), a chronic infectious and zoonotic disease caused by *Mycobacterium tuberculosis* complex (MTC), is considered a neglected disease of global importance, causing a detrimental impact on public health, particularly in developing countries where tuberculosis remains a major health problem. However, debate around the efficacy of control measures is still an ongoing matter of concern, with poor diagnostic performance being considered one of the most relevant factors involved in the failure to eradicate the disease since many truly infected animals will be misclassified as bTB-free. This study highlights a DNA extraction protocol and real-time PCR targeting IS6110 or IS4 as potential first-choice molecular assays to detect MTC directly in fresh bovine tissue samples, providing rapid, highly sensitive, and specific diagnostic tools as an alternative to microbiology, which could take up to 3 months to complete, shortening the turnaround time for decision makers to be promptly informed.

## INTRODUCTION

Bovine tuberculosis (bTB) is a chronic infectious and zoonotic disease caused mainly by not only *Mycobacterium bovis* and *Mycobacterium caprae* but also other members of the *Mycobacterium tuberculosis* complex (MTC) (1, 2). bTB is considered a neglected disease of global importance, causing a detrimental impact on public health, particularly in developing countries where tuberculosis is likely to remain a major health problem (3). In the European Union, bTB is subjected to obligatory eradication programs based on the detection of the cellular immune response by single or comparative intradermal tuberculin testing (SITT or CITT), gamma interferon testing, and surveillance in slaughterhouses of the presence of tuberculosis-like lessions (TBL), characterized by granulomatous lesions [Commission Delegated Regulation (EU) 2020/689]. Even though enormous sums of public funding are mobilized to ensure efficient surveillance systems and control programs, there are territories struggling to eradicate bTB at the herd-level in several European countries, where debate around the efficacy of these measures is an ongoing matter of concern. Thus, the failure to eradicate the disease could be explained by a combination of factors, though poor diagnostic performance is likely to be one of the most relevant since many truly infected animals will be misclassified as bTB-free. In the case of cattle, the main reservoir of MTC, these misclassified infected animals will not only contribute to keeping the chain of infection in the farm but also share pasture areas with other domestic and wild animals, which are likely to play a key role as reservoirs as well, hindering the success of bTB eradication programs.

Nowadays, selective microbiological culture is considered the gold standard technique for bTB diagnosis, with recovery rates ranging from 30% to 95% (4). Nevertheless, it has been proven that microbiological culture is also an imperfect technique, with sensitivity (SE) and specificity (SP) values of 78.10% (72.90%–82.80%) and 99.10% (97.10%–100.0%), respectively (4). In addition, this technique requires high biosecurity facilities, the technicians should hold relatively high expertise, and every bacterial growth needs to be confirmed by PCR (4, 5). Another point to take into consideration is the extremely slow growth of the members of MTC, requiring long incubation periods of up to 12 weeks (6), which delays the turnaround time to get a confirmatory result. Because of these weaknesses, it would be necessary to develop rapid, cost-effective, and accurate diagnostic tools, particularly in the current framework of the European bTB surveillance and control program, where macroscopic lesion disclosure at the slaughterhouse and prevalence have been decreasing over the last few years.

Accordingly, real-time PCR assays have been reported to be a rapid and accurate diagnostic tool, resulting in a potential first-line assay for the speedy and direct detection of MTC in fresh bovine tissue samples (7, 8). The DNA isolation protocol is a crucial first step in the real-time PCR pipeline, determining the amount and quality of isolated DNA. In the case of MTC, it is important to underline several intrinsic limitations and/or factors challenging a direct detection of MTC, such as a robust cell envelope, the paubacillary nature of this complex, or the extensive necrosis, fibrosis, and mineralization associated with TBLs (9 – 12). In addition, some inhibitors, including an excess of host DNA and organic compounds, can play an important role in impairing the performance of real-time PCR. These inhibitors could be avoided or accidentally introduced into the sample during the DNA extraction process. These limiting factors could have a negative impact on the yield and quality of isolated DNA and, consequently, the success of real-time PCR, leading to false-negative results.

Likewise, DNA regions selected as PCR targets could be a key player in the success of the PCR in diagnosis. Consequently, the IS*6110* sequence, which is found in multiple copies in pathogens belonging to MTC, is commonly used for PCR detection of MTC (4, 13 – 15). Nevertheless, cross-reactivity with certain primer pairs or probes has been previously reported (16). Besides IS*6110*, other targets have also been used for the same purpose with differing SE and SP results, including IS*1081* (10, 17), DevR (18), Ku gene (19), mpb83 (20), hupB (21, 22), 16 S-23S rRNA internally transcribed spacer (23), p34 gene (24), TbD1 (25) or IS*4* (26), and mpb70 (16) more recently.

According to these issues, not only IS*6110* but also mpb70, a gene encoding a highly specific and immunogenic protein conserved in all members of the MTC (27) and the insertion sequence IS*4*, a 141-base pair (bp) reported as one of the most highly conserved regions found in IS*6110* (26), represent potential DNA targets for MTC diagnosis by using real-time PCR in fresh tissue samples. Consequently, the goal of the present study was, first, to optimize and compare the performance of two DNA extraction protocols for MTC detection by real-time PCR using bovine fresh lymph nodes as input samples and, second, to evaluate and validate a real-time PCR targeting IS*6110*, mpb70, IS*4*, and/or a combination of these specific regions in comparison with microbiological culture in bTB eradication programs.

## Materials and Methods

### Sample selection and processing

This study was part of a larger project focused on improving rapid and accurate diagnostic tools in the framework of the bTB surveillance and control program in Spain. In brief, fresh lymph node (LN) tissue samples were collected from cattle carcasses at the slaughterhouse from 2018 to 2019 in the context of the Spanish bTB eradication program. All samples were collected during routine post-mortem veterinary examinations within an official context and according to national and European regulations. No purposeful killing of animals was performed for this study, so no ethical or farmer’s consent approval was required.

In order to perform this study, LN fresh tissue samples were collected from 81 animals, verifying the presence of either visible TBLs or non-visible lesions (NVLs). Individual homogenization of each LN was run using a tissue homogenizer (Fisherbrand, Fisher Scientific, Madrid, Spain) to obtain a uniform mixture. Tissue homogenate was split up into paired samples that were used for DNA isolation and selective microbiological culture, respectively.

### *Mycobacterium tuberculosis* complex microbiological culture

The samples were analyzed by the reference technique, microbiological culture, followed by PCR confirmation according to the previously described protocol (28). Briefly, the homogenate was decontaminated with an equal volume of 0.75% (wt/vol: 1/1) hexadecylpyridinium chloride solution in agitation for 30 min. Samples were centrifuged for 30 min at 1,500 × *g* (28). The pellets were collected with swabs and cultured in liquid media (MGIT 960, Becton Dickinson, Madrid, Spain) using an automated BD Bacter MGIT System (Becton Dickinson). The culture was considered positive when colonies were confirmed as MTC by real-time PCR (29).

### Optimization of DNA extraction protocols from homogenized fresh tissue lymph nodes

#### DNA extraction using a commercial kit (protocol 1)

DNA extraction using a commercial kit (protocol 1) from homogenized tissue samples was conducted using DNA Extract VK (Vacunek, Bizkaia, Spain) according to the manufacturer’s guidelines with several modifications. Briefly, a mix of 300 mg of homogenized tissue was submitted to mechanical disruption (30 Hz/20 min) together with 250 µL of sterile distilled water, 250 µL of sample lysis buffer VK-SB, and 300 mg of 0.5 mm glass beads. After that, tissue samples were centrifuged, the supernatant was discarded, and the sediment was subjected to an enzymatic digestion with proteinase K at 56°C in a thermo-shaker (750 rpm/12 h). Next, lysis buffer VK-LB3 was added, and the mixture was incubated for 10 min at 70°C. Finally, 210 µL ethanol (96%–100%) was added to the sample, which was applied in a spin column following the manufacturer’s guidelines.

#### Mechanical lysis, proteinase K digestion and DNA extraction using a commercial kit (protocol 2)

**FIG 1:**
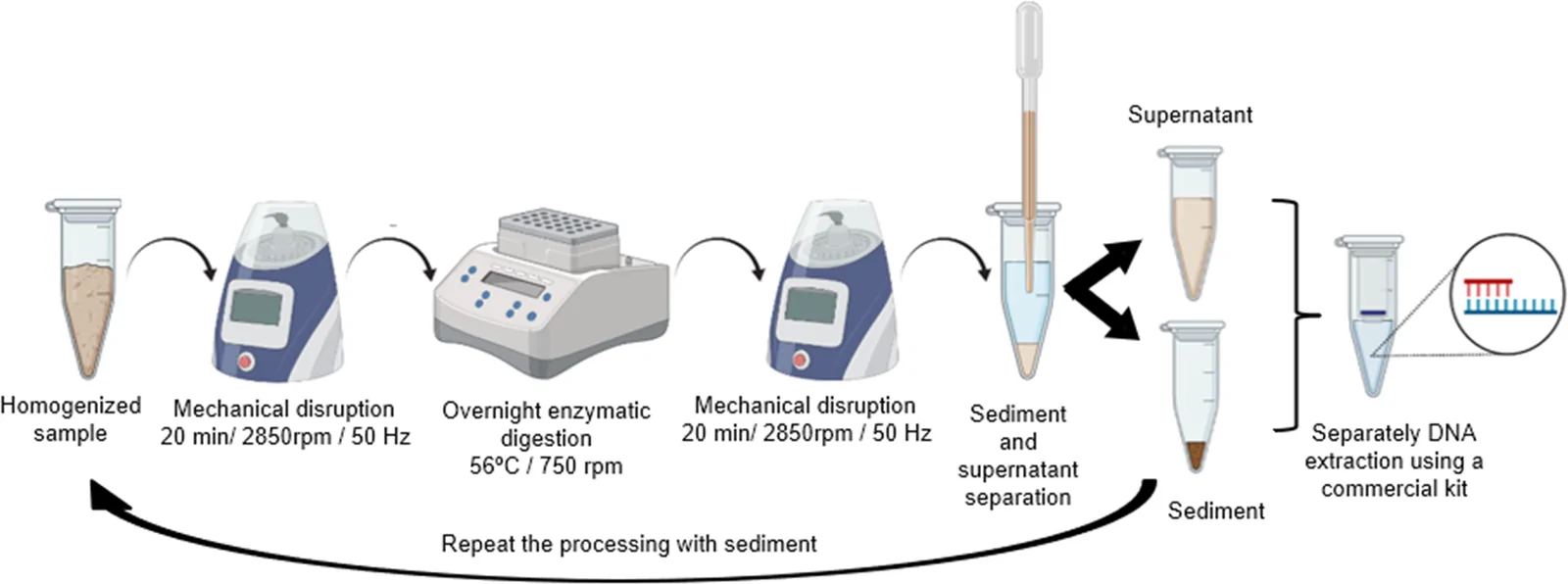
Flow chart of mechanical lysis, proteinase K digestion, and DNA extraction using a commercial kit (protocol 2).

Protocol 2, which consists of mechanical lysis, proteinase K digestion, and DNA extraction using a commercial kit (Fig. 1), was performed according to Lorente-Leal et al. (16) with several modifications by using NucleoSpin Tissue Kit (Macherey-Nagel, Düren, Germany). In brief, 1 mL of homogenized tissue (1,000 mg) was centrifuged for 5 min at 9,000 × *g*. The resulting tissue pellet was transferred into a tube together with 250 µL of sample buffer T1 and 150 mg of 0.5 mm and 50 mg of 0.1 mm glass beads. Then, samples were subjected to mechanical disruption using Scientific Industries SI Disruptor Genie (2,850 rpm/50 Hz/20 min). After an overnight enzymatic digestion at 56°C with 30 µL proteinase K in a thermo-shaker (750 rpm/12 h), a new mechanical disruption step was conducted. Samples were centrifuged for 2 min at 9,000 × *g*, and then the supernatant was transferred to a new tube, preserved to be processed afterward, while sediments were treated again with a new cycle of mechanical disruption and enzymatic digestion according to the steps of DNA extraction performed as outlined above in this protocol 2.

Then, both the sediment and supernatant of each sample were processed independently and mixed with 200 µL of Buffer T3, incubating the mixture for 10 min at 70°C. The lysate was transferred to a silica-based nucleic acid purification column and managed according to the manufacturer’s instructions. Positive and negative extraction controls were included for both protocols 1 and 2. All DNA extraction products were stored at −20°C until use.

### Optimization of real-time PCR from fresh tissue samples

Specific primers and probes targeting IS*6110*, IS*4*, and mpb70 were used for this study. Specific primers (IS*6110-forward*: 5′-GGTAGCAGACCTCACCTATGTGT-3′; IS*6110-reverse*: 5′-AGGCGTCGGTGACAAAGG-3′) and probes (IS*6110-probe*: 5′-FAM-CACGTAGGCGAACCC-MGBNFQ-3′) were selected from previous studies (7, 30). These oligonucleotides target a 68-bp region of the transposon IS*6110*, which is specific for MTC pathogens. For IS*4*, the primers (IS*4-forward*: 5′-CTCGACCTGAAAGACGTTATCC-3′ and IS*4-reverse*: 3′- CTCGGCTAGTGCATTGTCATA-5′) and the probe (IS*4-probe*: 5′-AGTACACAT/ZEN/CGATCCGGTTCAAGCG-3′), targeting a 141-bp highly conserved region in the transposon IS*6110*, were used (26). For mpb70 (16), the following oligonucleotides targeting a 133 bp region of the mpb70 gene, which encodes one of the most specific and immunogenic protein conserved in all members of the MTC (27) were used: mpb70*-forward:* 5′-CTCAATCCGCAAGTAAACC-3′; mpb70*-reverse:* 5′-TCAGCAGTGACGAATTGG-3′), and the probe (mpb70*-probe*: 5’- FAM-CTCAACAGCGGTCAGTACACGGT-BHQ1-3’).

QuantiFast Pathogen PCR +IC Kit (Qiagen, Hilden, Germany) was used to conduct the real-time PCR evaluating each sample in duplicate in the MyiQ2 Two-Color Real-Time PCR Detection System (Bio-Rad, Hercules, CA, USA) under the following cycling conditions: 95°C for 5 min to activate the DNA polymerase followed by 42 amplification cycles that consisted of a denaturation step at 95°C for 15 s and an annealing-extension step at 60°C for 30 s. Moreover, as the manufacturer’s guidelines described, an exogenous inhibition heterologous control [internal control assay (ICA)] was included to evaluate the presence of certain inhibitors in the samples. The setup for 10  µL of reaction volume was 4  µL of ultrapure distilled water, 1  µL of sample, 2 µL of Quantifast pathogen master mix, 1 µL of ICA, 1  µL of internal control DNA, and for IS*6110*, 0.4  µL of forward (10  pmol/µL), 0.4  µL of reverse (10  pmol/µL), and 0.2 µL of probe (10  pmol/µL), or for IS*4* and mpb70, 0.6 µL of forward (10  pmol/µL), 0.3 µL of reverse (10  pmol/µL), and 0.1 µL of probe (10  pmol/µL). The IAC Cq should be 30 ± 3.

Complete inhibition of amplification was considered when IAC did not amplify and partial inhibition of amplification when it showed a Cq value >33. When inhibition was detected, the samples were diluted 1:2, and the real-time PCR was run again. An inter-run calibrator with a known Cq value of 32.0 was introduced in each assay to self-control intra-assay repeatability and accuracy. In the case of culture-positive and real-time PCR-negative samples with a Cq value >38, DNA extraction and real-time PCR were repeated to verify the results. In the case of protocol 2, for every sample, PCR was first conducted from the DNA extraction obtained from the sediment. When a negative result was obtained, amplification was carried out using the DNA isolated from the supernatant as template.

### Limit of detection

The analytical sensitivity, or limit of detection (LOD), was estimated for the proposed primers and probes. LOD is defined as the lowest concentration at which 95% of replicates are positive, according to the Clinical and Laboratory Standard Institute guidelines. A serial 10-fold dilution series of *M. bovis* genomic DNA with known quantities ranging from 10^6^ to 10^0^ was used. The reactions were performed in triplicate for each dilution in three different assays.

### Statistical analysis

The performance of both DNA extraction protocols and real-time PCR targeting IS*6110*, IS*4*, and mpb70 was evaluated by comparing them with microbiological culture, which is considered an imperfect reference technique for bTB diagnosis (4, 5, 17). Using statistical Epidat 3.1 software (Galician Health Service, Spain), the adjusted SE and SP, false positive rate (FPR), and false negative rate (FNR) were estimated. In addition, positive and negative likelihood ratio (PLR and NLR) results were determined and interpreted following the criteria published below by (31): (i) PLR ≥10 or NLR ≤0.1, a technique of high diagnostic value that will normally allow discrimination between healthy and diseased animals; (ii) PLR = 5–10 or NLR = 0.1–0.2, a technique involving moderate changes in probability and whose diagnostic utility will depend on prevalence; (iii) PLR = 2–5 or NLR = 0.2–0.5, involving small changes in probability and whose diagnostic utility will depend on prior probability; and (iv) PLR = 1–2 and NLR ≤0.5, rarely discernible changes. Positive and negative predictive values (PPV and NPV) for different infection prevalences were subsequently estimated using a Bayesian approach (EPIDAT 3.1, Galician Health Service, Spain). Results were plotted using GraphPad Prism 9 (GraphPad Software, La Jolla, CA, USA).

The SE and SP for each DNA extraction protocol and each real-time PCR target, IS*6110*, IS*4,* or mpb70, the 95% confidence interval (CI), and the McNemar’s chi-squared test for correlated proportions in subgroups of MTC-infected and non-infected animals (H_0_ = equal detection proportion, *P* value <0.05) (32) (IBM SPSS Statistics version 28.0.1.1; IBM Corp.) were calculated. Finally, agreement between microbiological culture and real-time PCR targeting IS*6110*, IS*4*, or mpb70 was assessed using Cohen’s kappa coefficient (κ): κ = 0 indicated no agreement; 0.01 ≤ κ ≤ 0.20, slight agreement; 0.21 ≤ κ ≤ 0.40, fair agreement; 0.41 ≤ κ ≤ 0.60, moderate agreement; 0.61 ≤ κ ≤ 0.80, substantial agreement; and 0.81 ≤ κ ≤ 1.00, almost perfect agreement (33) (WinEpi software 2.0, Faculty of Veterinary Medicine, University of Zaragoza, Spain).

## RESULTS

### *Mycobacterium tuberculosis* complex microbiological culture results

Results of MTC microbiological culture were used as the reference assay for comparing extraction protocols 1 and 2. Consequently, a sample was considered a culture-positive sample when an obtained colony from a grown culture was confirmed by real-time PCR-IS*6110*. Thus, 49.38% of animals (*n* = 81) were disclosed as culture-positive (*n* = 40), whereas 50.62% were tested as culture-negative (*n* = 41). Besides, in order to disclose TBL, every LN was subjected to a gross evaluation, revealing 19.75% of animals with TBLs (*n* = 16), with 87.50% of them being culture-positive (*n* = 14) (Table 1).

**TABLE 1:** Description of the obtained results by microbiological culture, real-time PCR-IS*6110* (protocols 1 and 2)*,* IS*4*, and mpb70 in relation to the presence of tuberculosis-like lesions

	Overall comparison in relation to the presence or absence of TBL									
	Microbiological culture		IS*6110* (protocol 1)		IS*6110* (protocol 2)		IS4		Mpb70	
	+^*a*^	^*b*^−	+	−	+	−	+	−	+	−
TBL^*c*^	14	2	16	0	16	0	16	0	15	1
NVL^*d*^	26	39	17	48	29	36	27	38	24	41
Total	40	41	33	48	45	36	43	38	39	42

^*a*^ +, positive.

^*b*^ −, negative.

^*c*^ TBLs, tuberculosis-like lesions.

^*d*^ NVLs, non-visible lesions.

### Optimization of DNA extraction protocols from homogenized fresh tissue lymph nodes by using IS*6110* target

The DNA isolation protocol is a critical first step in the real-time PCR pipeline. In order to identify a DNA extraction method that yields suitable genomic DNA in terms of quality and amount, we tested two different DNA extraction protocols. The performance of both extraction protocols was evaluated by running a real-time PCR targeting IS*6110*, as previously described by our laboratory, with LOD ranges from 10 to 100 genomic equivalents and the cut-off set at Cq <38.

#### MTC real-time PCR-IS6110 results according to DNA extraction by protocol 1

Eighty-one animals were evaluated by protocol 1, with Cq values ranging from 24.60 to 37.50 (average = 30.20) after real-time PCR-IS*6110* (Table 1). Thus, 40.74% of animals were tested as MTC-positive (*n* = 33), and 59.26% were tested as MTC-negative (*n* = 48). The IAC amplified in most of the samples without partial inhibition, but when complete inhibition was observed because of a high yield of DNA (over 1,000 ng/µL), samples (*n* = 6) were diluted up to a final concentration of 450 ng/µL and re-evaluated by real-time PCR, keeping a negative result for MTC but with IAC amplification. Taking into consideration TBLs, 100% of LN samples with TBLs that were evaluated by protocol 1 were tested as MTC-positive (*n* = 16 animals) by real-time PCR-IS*6110* (Table 1).

#### MTC real-time PCR-IS6110 results according to DNA extraction by protocol 2

In the case of extraction protocol 2, 55.56% of all analyzed animals were tested as MTC-positive (*n* = 45), and 44.44% were tested as MTC-negative (*n* = 36) (Table 1). Whereas 86.67% of MTC-positive samples (*n* = 39) came from the sediment, 13.30% of MTC-positive samples (*n* = 6) were finally detected from the supernatant after obtaining negative or inconclusive results from the sediment (data not shown). The Cq values ranged from 21.00 to 37.50 (average = 32.91) for sediments and from 27.00 to 37.00 (average = 32.67) in the case of supernatants. Although IAC amplification was observed in most of the samples, several partial (*n* = 10) or complete inhibitions (*n* = 14) were found. Then, samples were diluted 1:2 and re-evaluated by real-time PCR. Five samples were detected as positive, and the remainder kept negative results for MTC but with IAC amplification. All the samples with TBLs that were evaluated by protocol 2 were tested as MTC-positive (*n* = 16 animals) by real-time PCR-IS*6110* (Table 1).

#### DNA extraction protocols 1 and 2: validation and comparison

Taking into consideration that microbiological culture is considered an imperfect assay for performing bTB diagnosis (4, 5, 17), the diagnostic performance results, summarized in Table 2, were calculated for an imperfect gold standard using EPIDAT 3.1 software. In the case of DNA extraction protocol 1, 31 out of 40 animals positive to microbiological culture were also classified as MTC-positive by real-time PCR-IS*6110* [adjusted SE = 78% (95% CI: 65%–91%)], while 39 out of 41 culture-negative animals resulted in being negative by real-time PCR-IS*6110* [adjusted SP = 100% (95% CI: 100%)], with an adjusted FNR and FPR of 21.70% (95% CI: 9%–35%) and 0% (95% CI: 0%), respectively. Based on the positive and negative likelihood ratios (PLR = *∞* and NLR = 0.22) (Table 2), protocol 1 would have a high diagnostic value for the positive results, normally allowing discrimination between healthy and diseased animals, while the diagnostic utility of negative results would be dependent on the prior probability of TB in the area. The agreement with microbiological culture was substantial (κ = 0.72).

**TABLE 2:** Diagnostic performance summary of real-time PCR-IS6110 by comparing with microbiological culture assays under two different extraction protocols, 1 or 2^*f*^

Diagnostic accuracy (95% CI)^*e*^						
	Sensitivity	Specificity	FPR^*a*^	FNR^*b*^	PLR^*c*^	NLR^*d*^
IS6110 (extraction protocol 1)	78.30% (65.40%–91.20%)	100% (100%)	0% (0%)	21.70% (8.80%–34.60%)	∞	0.22
IS6110 (extraction protocol 2)	95.90% (89.70%–100%)	100% (100%)	0% (0%)	4.10% (0%–10.30%)	∞	0.04

^*a*^ FPR: false positive ratio.

^*b*^ FNR: false negative ratio.

^*c*^ PLR: positive likelihood ratio.

^*d*^ NLR: negative likelihood ratio.

^*e*^ 95% CI: 95% confidence interval.

^*f*^ Estimates were calculated for an imperfect gold standard assay using EPIDAT 3.1 software (Software for Epidemiologic Analysis of Tabulated Data).

By contrast, in the case of DNA extraction protocol 2, 38 out of the 40 animals positive to culture were classified as MTC-positive [adjusted SE = 96% (95% CI: 90%–100%)], while 34 out of the 41 culture-negative animals were classified as MTC-negative [adjusted SP = 100% (95% CI: 100%)]. It is important to mention that 7 animals negative to culture were disclosed as positive (2 out of 7 were confirmed to present TBLs), while we found 2 culture-positive samples without TBLs but PCR-IS*6110-*negative. Consequently, the adjusted FNR of 4% (95% CI: 0%–10%) and FPR of 0% (95% CI: 0%) were estimated (see Table 2). According to the values estimated for LR (PLR >10 and NLR = 0.04), this protocol demonstrated a high diagnostic utility to confirm and discard bTB regardless of its true prevalence. The agreement with microbiological culture was substantial (κ = 0.77).

The Mcnemar test was used to compare both protocols, finding a statistically significant difference (*P* = 0.016) between the number of culture-positive animals detected using DNA extraction protocol 2 compared with protocol 1. No differences were found for culture-negative animals between both protocols.

### Validation and diagnostic performance of real-time PCR targeting IS*4* and mpb70

According to these results, DNA from samples processed by protocol 2 was further analyzed by real-time PCR targeting IS*4* or mpb70, comparing these results with microbiological culture to validate these targets for MTC detection by real-time PCR from fresh cattle tissue samples.

#### Real-time PCR targeting IS4

Forty-three out of 81 animals (53.10%) were disclosed as positive by real-time PCR-IS*4*, and 38 were tested as MTC-negative (46.90%). All the samples with TBLs (*n* = 16 animals) were tested as PCR-IS*4*-positive (Table 2). The analysis of the sediment disclosed 86.05% of MTC-positive samples (*n* = 37), while 13.95% of MTC-positive samples (*n* = 6) yielded a positive result from the supernatant. The Cq values ranged from 22.96 to 38.10 (average = 32.06) for the sediment, and, in the case of the supernatant, the Cq values ranged from 26.00 to 37.00 (average = 32.87). A partial inhibition of IAC was found in 5 out of 81 samples, probably due to the presence of some inhibitors, with 2 out of 5 samples disclosing a positive result after dilution 1:2 and re-evaluation. The LOD for this real-time PCR-IS*4* ranged from 50 to 100 genomic equivalents, and the cut-off was established at Cq <39.

Comparing with the microbiological culture as the reference assay, 36 out of 40 animals positive to culture were also found to be positive to real-time PCR-IS*4* [SE adjusted = 91% (95% CI: 82.00%–99.80%)], and 34 out of 41 culture-negative animals were also tested as real-time PCR-IS*4-*negative [SP adjusted = 100% (95% CI: 100%)]. Noteworthy, seven culture-negative animals that were PCR-IS*6110-*positive were also disclosed as positive by real-time PCR targeting IS*4*. According to these results, the protocol would have an adjusted FNR of 9% (95% CI: 0.20%–18.00%) and an adjusted FPR of 0% (95 CI: 0%). Regardless of the true prevalence, real-time PCR-IS*4* was found to have a high diagnostic utility to confirm and discard MTC (PLR >10 and NLR = 0.09) (Table 3), with similar values to real-time PCR-IS*6110*. The concordance with microbiological culture (κ = 0.72) was substantial.

**TABLE 3:** Diagnostic performance summary of real-time PCR-IS6110, IS4, and Mpb70 compared with reference microbiological culture assays (imperfect gold standard assays) under the same cycling conditions^*f*^

Diagnostic accuracy (95% CI)^*e*^						
Target	Sensitivity	Specificity	FPR^*a*^	FNR^*b*^	PLR^*c*^	NLR^*d*^
IS6110	95.90% (89.70%−100%)	100% (100%)	0% (0%)	4.10% (0%–10.30%)	∞	0.04
IS4	91% (82.0%–99.80%)	100% (100%)	0% (0%)	9.10% (0.20%–18.0%)	∞	0.091
Mpb70	83.30% (71.60%–95.0%)	100% (100%)	0% (0%)	16.70% (5.0%–28.4%)	∞	0.16

^*a*^ FPR: false positive ratio.

^*b*^ FNR: false negative ratio.

^*c*^ PLR: positive likelihood ratio.

^*d*^ NLR: negative likelihood ratio.

^*e*^ 95% CI: 95% confidence interval.

^*f*^ Estimates were calculated for an imperfect gold standard using EPIDAT 3.1 software (Software for Epidemiologic Analysis of Tabulated Data).

#### Real-time PCR targeting mpb70

In the case of real-time PCR-mpb70, 39 of 81 tested animals were detected as MTC-positive (48.14%) and 42 as negative (51.86%). Thus, 35 samples positive for real-time PCR-mpb70 (89.74%) were disclosed from the sediment and 4 from the supernatant (10.25%). Besides, 100% of lymph nodes with TBLs (*n* = 16 animals) were tested to be PCR-mpb70 positive (Table 2). The Cq values ranged from 21.83 to 36.80 (average = 31.5) for the sediment and from 27.90 to 35.0 (average = 30.36) for the supernatant. As mentioned above for the other targets, because of some inhibitors, amplifications were found to be partially inhibited in 5 out of 81 samples, which were diluted 1:2 and re-evaluated, allowing the detection of 3 inhibited samples as positive. The LOD for real-time PCR-mpb70 was determined to be lower than 100 genome equivalents, and the cut-off was set to a Cq value of <38.

The real-time PCR targeting mpb70 detected 33 out of 40 culture-positive animals [SE adjusted = 83.30% (95% CI: 71.60%–95.00%)], and 35 out of 41 culture-negative animals resulted in being mpb70-negative [SP adjusted = 100% (95% CI: 100%)]. Of note, 6 out of the 7 culture-negative animals but positive to IS*6110* and IS*4* targets were also classified as MTC-positive by real-time PCR targeting mpb70. Thus, an adjusted FNR of 16.70% (95% CI: 5.00%–28.40%) and an FPR of 0% (95% CI: 0%) were estimated (see Table 3). The PLR value (PLR = *∞*) implies a high diagnostic value for the positive results discriminating between MTC-infected and uninfected animals; however, the NLR value was 0.16, meaning that the true prevalence (TP) of the area could impact the diagnostic utility of negative results for this assay (Table 3).

### Comparison between real-time PCR targets to detect MTC

In order to statistically evaluate the differences observed in the SE and SP of the real-time PCR depending on the DNA target, the 95% CI and correlated proportions of McNemar’s test were estimated (Table 4). No significant differences were found between DNA targets (*P* > 0.05). Cohen’s kappa coefficient (κ) showed an almost perfect agreement between all the targets (data not shown): IS*6110*-IS*4* (κ = 0.95), IS*6110*-mpb70 (κ = 0.90), and IS*4*-mpb70 (κ = 0.93).

**TABLE 4:** McNemar test^*a*^

	IS4					Mpb70					IS4				
		IS*6110*	+	−	McNemar		IS*6110*	+	−	McNemar		Mpb70	+	−	McNemar
Microbiological culture	Positive (*n* = 40)	+	36	2	0.5	Positive (*n* = 40)	+	33	5	0.1	Positive (*n* = 40)	+	33	0	0.3
		−	0	2			−	0	2			−	3	0	
	Negative (*n* = 41)	+	7	0	1.0	Negative (*n* = 41)	+	6	1	1.0	Negative (*n* = 41)	+	6	0	1.0
		−	0	34			−	0	34			−	1	34	

^*a*^ Pairwise frequencies of real-time PCR results for IS6110, IS4, and mpb70 in 81 animals according to microbiological culture results (positive *n* = 40 and negative *n* = 41).

**FIG 2:**
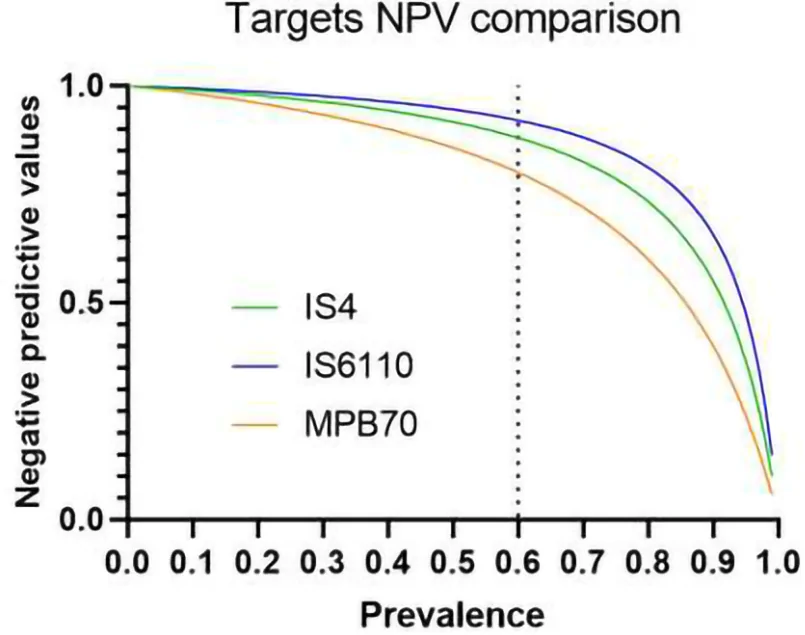
Graphical representation of the estimated NPV based on the validity of the real-time PCR targeting IS*6110* (blue line), IS4 (green line), and mpb70 (orange line) and the true prevalence of bTB.

In addition, the diagnostic utility of the positive and negative results obtained with each probe was compared based on true prevalence (predictive values). When the TP ranges from 0% to 60% (Fig. 2), the NPV for real-time PCR targeting IS*6110* is ≥90%, NPV ≥85% for IS*4*, and NPV ≥80% for mpb70. According to PPV (100%), real-time PCR targeting IS*6110*, IS*4*, and mpb70 is able to confirm bTB infection in any scenario of true prevalence.

## DISCUSSION

bTB is one of the most relevant animal diseases worldwide and, hence, a major concern for public health. Even though eradication is the main goal for the EU, this neglected zoonosis is still present in dairy and cattle herds, especially in several European regions. Nowadays, the approved surveillance systems, which are mainly based on cellular immune reactions, have facilitated the diagnosis of infected cattle at the early stage of the disease; therefore, animals with clinical signs or gross post-mortem TBLs are lacking or rarely found at the slaughterhouse (34, 35). This success of surveillance systems has challenged the direct detection of MTC or its DNA using microbiological culture and/or PCR since *in-vivo* immune responses have to be confirmed post-mortem (36). The selective microbiological culture is the gold standard technique for confirming bTB diagnosis, although it is considered an imperfect assay (4, 5, 17). Therefore, the development and validation of sensitive and specific real-time PCR protocols, including sample processing and DNA extraction steps, as an alternative to microbiological culture is likely to pave the way to bTB diagnosis. The present study aimed to evaluate two different DNA isolation protocols and three different specific DNA targets, IS*6110*, IS*4*, and mpb70, to confirm MTC infection by real-time PCR directly from fresh LN tissue samples.

DNA isolation is an essential step in the real-time PCR pipeline; therefore there are no standardized protocols to isolate mycobacterial DNA from fresh post-mortem samples, and different approaches can be found among studies (4, 16, 23, 37, 38). Mycobacteria belonging to MTC have some distinctive features that could potentially hinder diagnostic performance, including the characteristics of the MTC cell wall, a non-homogeneous distribution and intracellular location of MTC-bacillus, the well-known paucibacillary nature of this complex, and early infected animals with NVLs at the slaughterhouse (9 – 12). In order to solve these issues, a novel DNA extraction protocol (protocol 2) was compared with a traditional one (protocol 1) and microbiological culture, revealing promising results with the real-time PCR-IS*6110*. To do so, protocol 2 used a larger amount of tissue sample (300 mg for protocol 1 vs 1,000 mg for protocol 2), as well as additional mechanical disruption and enzymatic digestion steps. Accordingly, protocol 2 disclosed 38 out of 40 culture-positive samples (95%) and 7 culture-negative samples (2 out of 7 showed TBLs) as PCR-IS*6110*-positive. Furthermore, several animals with a negative result in protocol 1 turned out to be positive (*n* = 12) when protocol 2 was performed. Consequently, and according to the literature (16, 38), these results point out that the use of a small amount of tissue sample seems to decrease the chances of targeting mycobacterial DNA, leading to false-negative results, particularly in the case of infected animals with NVL.

Second, the incubation time before conducting the mechanical lysis step and the kind of disruption procedure have been reported to potentially impact downstream real-time PCR applications after DNA isolation, reducing the number of discordant results (16, 38, 39). Therefore, real-time PCR-IS*6110* showed higher diagnostic efficiency in our study after conducting protocol 2 than protocol 1 because tissue samples were submitted to a more intense lysis during the DNA extraction process, including four mechanical disruptions and two overnight chemical lysis steps with proteinase K. In addition, protocol 2 allows analyzing both the sediment and the supernatant, increasing the diagnostic sensitivity of the technique. As a result, in our study, 39 animals were detected as positive from the sediment samples, and the re-analysis of negative and inconclusive samples allowed the detection of 6 additional PCR-positive animals from the supernatant. To optimize protocol 2, the probability of pooling sediment and supernatant from the same sample was taken into consideration, thereby avoiding the need for two separate reactions. However, this step may have the drawback of diluting the samples. Consequently, samples with a Cq close to the detection limit may yield negative results.

Another significant point to consider is the presence of several inhibitors associated with the extraction protocol or an excess of host DNA that could lead to false negative results and therefore decrease sensitivity as a consequence of a partial or total inhibition of amplification, impacting negatively on the performance of the real-time PCR diagnosis. An exogenous heterologous IAC supplied by the manufacturer, which enjoys several advantages over endogenous or exogenous homologous IACs, was used to identify inhibitors (16) and allowed the detection of partial or total inhibition phenomena. Thus, 6 samples were found to be completely or partially inhibited for protocol 1, whereas 24 were for protocol 2. After diluting these samples, 5 out of the 30 inhibited samples became positive, underscoring the relevance of including an exogenous heterologous IAC.

Overall, diagnostic SE and SP for protocol 2 and PCR-IS*6110* were very good when compared to microbiological culture: 95.93% (95% CI: 89.70%–100%) and 100% (95% CI: 100%), respectively. By contrast, protocol 1 and PCR-*IS6110* DNA yield an adjusted diagnostic SE of 78.30% (95% CI: 65.40%–91.20%) and SP of 100% (95% CI: 100%).

Previous studies targeting IS*6110* (14, 16) have reported quite similar SE and SP results than those herein reported for protocol 2. In addition, it is noteworthy to remark that in those studies, there was a high proportion of the evaluated samples with TBL (from 39% to 57.81% of the total samples vs 19.80% in our study), and hence, these animals should undergo an advanced stage of bTB infection. These results mean that both protocols work as a suitable tool to confirm bTB in reactor animals with either TBLs or NVLs, but especially for bTB diagnosis under current field conditions in the case of protocol 2, which presented a higher sensitivity to diagnose reactor animals without evident gross lesions at the slaughterhouse. In addition, our results show that the extraction protocol is a definitely relevant step directly influencing the diagnostic SE of real-time PCR from fresh tissue samples.

On the other hand, not only does the DNA extraction method play a critical role in the detection of MTC but also the selection of the genetic target (12). The MTC-specific IS*6110* transposon is reported as one of the main targets of election for the diagnosis of the MTC complex by real-time PCR (40), providing a tool capable of differentiating between MTC and other bacteria, including non-tuberculous mycobacteria (NTM). However, an IS*6110*-like element has been recently found in the genome of other NTM, which may also potentially cross-react with certain IS*6110* primer pairs or probes (7, 13, 33, 41). Although this finding is expected to have a minimal impact on the specificity of the real-time PCR-IS*6110*, cross-reactivity cannot be ruled out, and the use of additional MTC-specific targets would be desirable to improve the diagnostic performance of direct real-time PCR from tissue samples. Thus, DNA isolated by protocol 2 was evaluated in the present study using two additional different genetic targets, IS*4* and mpb70. IS*4* DNA target, described by Wang et al. (26) on human clinical samples but never tested for veterinary diagnostics, is a highly conserved region inside IS*6110* found exclusively within the MTC. The *Mpb70* gene encodes an antigenic protein that is highly expressed by all members of the MTC, but it is a single-copy gene. The IS*4* and mpb70 real-time PCR, in combination with the obtained DNA with the extraction protocol 2, resulted in a rapid technique with good diagnostic performance [SE, 91% (82%–99.80%), and SP, for IS*4*; SE, 83.30% (71.60%–95%), and SP, 100% for mpb70] compared to microbiological culture. Considering previous real-time PCR studies (4, 14, 16, 24, 26), the estimated diagnostic SE for both DNA targets, IS*4* and mpb70, was significantly higher or similar to that previously reported, along with a higher estimated SP. Nevertheless, an individual comparison between studies could be problematic and definitely biased due to differences in molecular targets, DNA extraction protocols, and validation methods (42). Moreover, we found a substantial agreement between microbiological culture and real-time PCR-targeting IS*6110* (κ = 0.77), IS*4* (κ = 0.72), or mpb70 (κ = 0.67).

Even if SE and SP are the main diagnostic performance indicators used, there are other factors that could influence the final utility of a technique, such as the disease’s true prevalence in the region, the available laboratory resources, or its acceptability by veterinary professionals. The predictive values allow to estimate the variations in the diagnostic utility of a test based on its prevalence, so these values have great importance in allowing veterinary practitioners to interpret their results (43). Based on the obtained PLR and NLR, a real-time PCR targeting IS*6110*, IS*4*, or mpb70 positive results would confirm the bTB infection in any true prevalence scenario with 100% security (PPV). Regarding the credibility of the negative results, it was estimated an NPV of 90%, 85%, and 80% for real-time PCR targeting IS*6110*, IS*4*, and mpb70 respectively, when the true prevalence ranged from 0% to 60%. These results highlight the usefulness of IS*6110*, IS*4*, and mpb70 as targets of interest in the direct molecular diagnosis of bTB infection.

Despite the described results, several contradictory results between microbiological cultures and real-time PCR assays were observed. Of the 41 culture-negative animals, MTC DNA was detected in 5 animals with PCR targeting both IS*6110* and IS*4*, whereas PCR targeting mpb70 detected 4 animals as positive. These findings could be associated with different factors impacting the sensitivity and/or specificity of microbiological culture and real-time PCR. Among the limitations of microbiological culture impacting MTC detection, a lack of analytical specificity due to the growth of other microorganisms, including NTM (4, 5), or a decontamination process reducing the cell viability of a slow-growing bacteria with a paubacillary presentation (5, 9, 10, 12) should be taken into account. Furthermore, cross-reactivity of the probes with other bacteria should be considered a limitation of real-time PCR. As mentioned above, although IS*6110* is commonly used for the diagnosis of bTB, it may present cross-reactivity with NTM. To address this issue, IS*4* and mpb70 primer pairs, which have been reported to be MTC-specific (7, 44), were used in our study, proving that non-cross-reaction was found in culture-negative but real-time PCR-IS*6110*-positive animals. Besides, 2 out of 41 culture-negative animals and real-time PCR targeting IS*6110*, IS*4*, and mpb70-positive animals presented TBL confirmed by histopathology (data not shown). These results suggest that DNA extraction protocol 2 working together with real-time PCR targeting IS*6110*, IS*4*, or mpb70 could be a faster and more efficient assay for MTC detection in TBL or NVL samples during official post-mortem inspection compared to microbiological culture. Nevertheless, microbiological culture has actually been an essential technique for mycobacterial isolation and molecular epidemiology studies so far (4).

On the other hand, a small number of animals positive to microbiological culture were found to be PCR-negative targeting IS*6110* (2 out of 41), IS*4* (4 out of 41), or mpb70 (7 out of 41). The presence of inhibitors impairs real-time PCR performance; nevertheless, this factor was ruled out since IAC amplifications were observed in all of these PCR-negative samples. Another reason that could explain these negative results could be a low mycobacterial load that would be beyond the LOD, resulting in undetectable results for these assays, especially in the case of mpb70, which is a single-copy gene (16). In addition, although it seems to be an uncommon case, several isolates lacking the IS*6110* element have been reported not only for *M. tuberculosis* (45 – 47) but also for *M. bovis* (48).

The present study describes a complete protocol, including sample pre-processing, DNA purification, and real-time PCR analysis. According to our results, DNA extraction protocol 2 and real-time PCR targeting IS*6110* or IS*4* could be potential first-choice molecular assays to detect MTC directly in fresh bovine tissue samples. These protocols proved to be rapid, highly sensitive, and specific diagnostic tools as an alternative to microbiological culture, which could take up to 3 months to complete, shortening the turnaround time for decision makers to be promptly informed. Furthermore, the low proportion of animals tested with TBL (16 out of 81) in our study highlights the diagnostic potential of this real-time PCR protocol to detect MTC directly from fresh LN tissue samples at early stages of infection and, therefore, when the mycobacterial load is low. This would be essential in the current framework, in which successful eradication schemes have reduced the number of reactors with TBL at the slaughterhouse; thus, the implementation of these cost-effective molecular tools in surveillance and control programs would pave the way to eradicate bTB. However, the major limitation of this study that hinders its wide application in the present setting is the lack of a ring trial that would allow its validation in different laboratories as well as different epidemiological scenarios. Therefore, further work on the re-validation of the present protocol should be performed in the future.
